# Association between 24-hour movement behaviors and perceived stress in Chinese university students: a compositional data analysis

**DOI:** 10.3389/fpsyg.2025.1681198

**Published:** 2025-10-30

**Authors:** Defeng Dong, Yanhe Qu, Dianbo Zhang, Chen Dong

**Affiliations:** ^1^School of Sport Management, Shandong Sport University, Jinan, China; ^2^School of Physical Education and Sports, Central China Normal University, Wuhan, China

**Keywords:** physical activity, sedentary behavior, sleep, isotemporal substitution, perceived stress

## Abstract

**Objective:**

This study used objectively measured data and compositional data analysis to examine the relationship between 24-hour movement behaviors and perceived stress in Chinese university students.

**Methods:**

Cross-sectional data were collected from 208 Chinese university students (mean age = 20.23 years, 52.9% female). Accelerometers were used to measure 24-hour movement behaviors, including moderate-to-vigorous physical activity (MVPA), light physical activity (LPA), sedentary behavior (SB), and sleep. The Perceived Stress Scale (PSS-14) assessed perceived stress. Compositional data methods were applied to analyze the relationship between the proportion of time spent in 24-hour activities and perceived stress.

**Results:**

Compositional regression analysis indicated that time spent in MVPA (*β* = −2.55, *p* < 0.05) and LPA (*β* = −3.39, *p* < 0.05) was inversely associated with perceived stress, while SB (*β* = 7.95, *p* < 0.05) was positively associated. Sleep time (*β* = −2.29, *p* > 0.05) was not significantly related to perceived stress. Isotemporal substitution models further showed that replacing SB with MVPA, LPA, or sleep was associated with significant reductions in perceived stress scores, whereas reallocating time from MVPA, LPA, or sleep to SB was linked to significant stress increases.

**Conclusion:**

The proportion of time spent in MVPA and LPA was negatively associated with perceived stress among university students. Replacing sedentary behavior with MVPA or LPA was associated with lower perceived stress. However, these findings should be interpreted with caution due to the study’s cross-sectional design and reliance on self-reported sleep data.

## 1 Introduction

Stress can be defined as a state of worry or mental tension triggered by challenges or difficulties (Tavolacci et al., 2013). In small doses or over short durations, stress may serve as a motivator, enhancing resilience. However, acknowledging the psychophysiological complexity of stress reveals that its impact extends beyond the mental realm, involving intricate interactions between the brain, body, and hormonal systems. Chronic stress, for instance, can lead to maladaptive physiological responses, including dysregulation of the autonomic nervous system and prolonged cortisol release, which can have detrimental effects on both physical and mental health (McEwen, 2008; World Health Organization, 2023). Research has demonstrated that higher stress levels are linked to cardiovascular disease (Kivimäki et al., 2023), and early exposure to high stress increases the risk of cardiovascular disease, substance abuse, and psychiatric disorders by approximately two- to six-fold in adulthood (Kivimäki and Steptoe, 2018). A large-scale study of 485,000 participants found that, over an eight-year follow-up, severe psychological stress was associated with a 1.5-fold increased risk of mortality (Momen et al., 2020).

The university years represent a critical developmental stage, marking the transition from late adolescence to adulthood. During this period, students typically face multiple challenges from academic and social domains, requiring adaptation to new learning environments and social roles, which may lead to significant psychological stress. Notably, contemporary Chinese university students face distinctive sociocultural pressures commonly referred to as “involution.” The term denotes an extremely intense competitive social environment in which individuals are compelled to undertake inhumanly long hours of study or work, thereby generating considerable tension and stress (Gu and Mao, 2023). This fierce internal competition for limited resources (e.g., job opportunities, places in graduate programs) leads students to endure substantial academic pressure and psychological distress, which are major factors contributing to increased perceived stress and sedentary behaviors such as prolonged periods of study.

Prior evidence suggests that lifestyle modifications, including regular physical activity (PA), improved sleep, and mindfulness, are associated with reduced perceived stress (Stults-Kolehmainen and Sinha, 2014; Badger et al., 2019; Firth et al., 2020). Research indicates that PA is linked to dampened stress-related neural activity, partially explaining its cardiovascular benefits (Nowacka-Chmielewska et al., 2022). Moderate-to-vigorous physical activity (MVPA) has been shown to enhance stress coping and recovery (Gerber et al., 2017; Wang H. et al., 2023; Li et al., 2024). Past studies have found that after 20–30 min of aerobic exercise, individuals report feeling calmer, and this calming effect can persist for hours after exercise (Jackson, 2013). Consequently, physical activity is widely recommended as a strategy for stress management (Tan et al., 2020). Additionally, growing research has linked sedentary behavior (SB) with stress, suggesting SB as a predictor of both metabolic risks and mental health outcomes (Huang Z. et al., 2022). Few studies have explored the relationship between SB and perceived stress among university students, but available evidence indicates that greater sedentary time is associated with higher perceived stress levels (Lee and Kim, 2018; Ge et al., 2020; Huang and Li, 2025).

However, existing research has several limitations. First, most studies focus on a single behavioral factor (e.g., MVPA or SB), failing to capture the holistic distribution of 24-hour movement behaviors in relation to perceived stress. For example, the allocation of time to PA, SB, and sleep may co-vary to influence stress levels (Pedišić et al., 2017; Dumuid et al., 2018). Second, previous studies have overlooked the temporal continuity and complementarity of daily activities. In reality, daily behaviors are competitive in nature (e.g., prolonged sedentary time displaces physical activity), and such patterns may be key determinants of stress perception. Third, much of the current literature relies on self-reported questionnaires, lacking analyses based on daily movement logs or objective data.

To overcome these limitations, compositional data analysis (CoDA) offers a more appropriate statistical framework (Dumuid et al., 2018). Since the time spent in all behaviors within a 24-hour day sums to a constant, they are inherently co-dependent. CoDA properly addresses this compositional nature, avoiding the spurious correlations and interpretational issues that can arise from standard regression analyses (Dumuid et al., 2019). Therefore, this study aimed to comprehensively examine the relationship between 24-hour movement behaviors and perceived stress among Chinese university students. We hypothesized that, after controlling for relevant confounders, the proportion of time spent in SB would be positively associated with perceived stress, while the proportions of time in MVPA and LPA would be negatively associated with perceived stress.

## 2 Materials and methods

### 2.1 Study design and participants

This cross-sectional study was conducted between October and December 2024. Convenience sampling was used to recruit university students in Shandong Province, China, via social media announcements. Inclusion criteria required participants to be: (1) first- to fourth-year undergraduate students, and (2) physically healthy, with no contraindications to exercise or physical/mental disorders. The study protocol adhered to the Declaration of Helsinki and its subsequent amendments. All participants provided written informed consent. This study was approved by the Ethics Committee of Shandong Sport University (Number: SD2024032).

**Figure 1:**
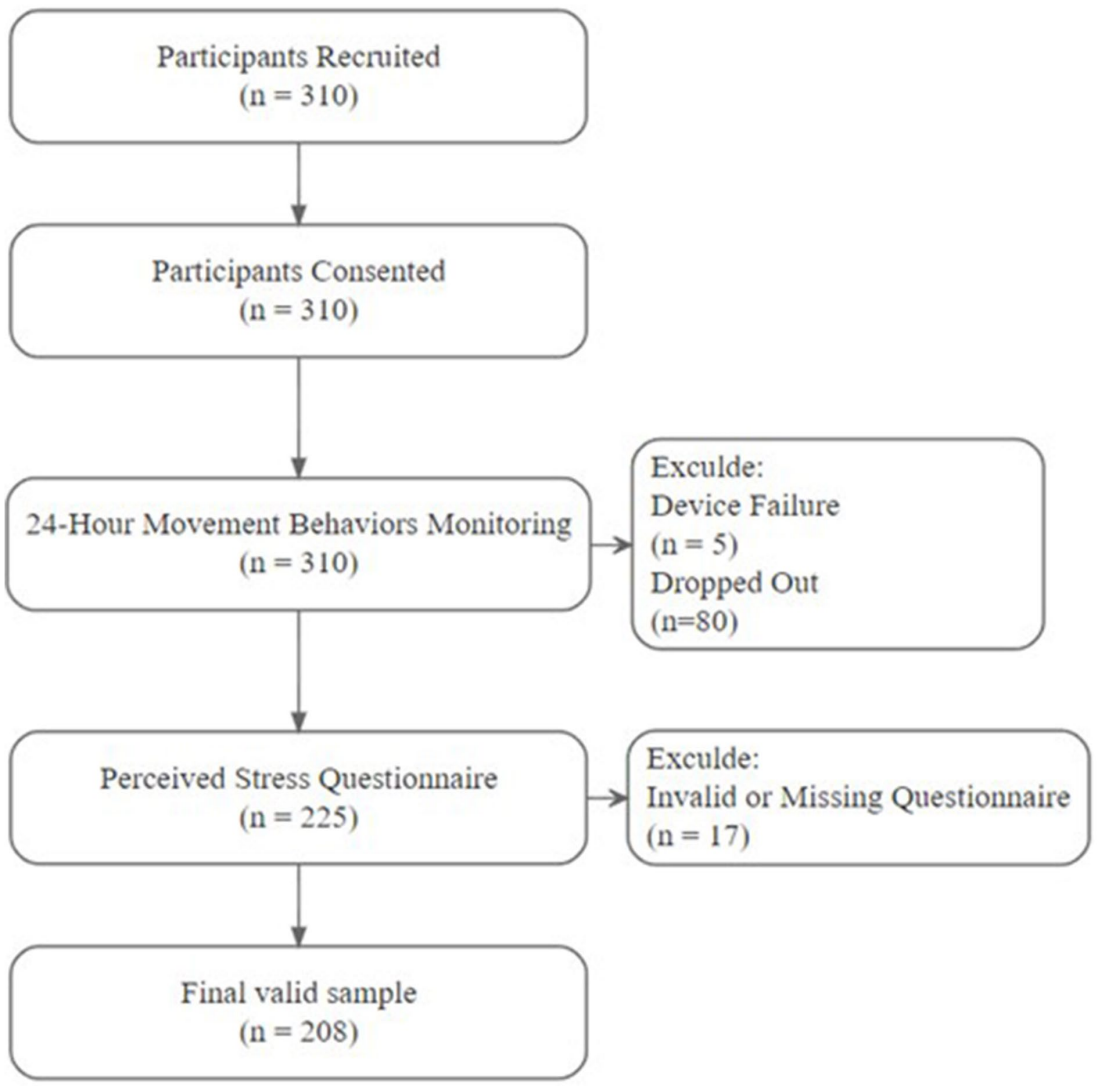
Flowchart of participant selection.

A total of 310 potential participants were initially recruited and provided consent. During the 24-hour movement behaviors monitoring phase, 53 individuals were excluded for failing to complete testing (five due to device failure, 80 dropped out). Subsequently, 225 participants completed the perceived stress questionnaire, but 17 were excluded for invalid or missing data. Ultimately, 208 participants were included in the final analysis (Figure 1). To evaluate whether this sample size affords adequate statistical power, sample size calculations were performed using GPower 3.1. For a multiple linear regression omnibus test (fixed model, *R*^2^ deviation from zero) assuming a medium effect size (*f*^2^ = 0.15), six predictors (for example: sleep, sedentary time, LPA, MVPA, age, sex), *α* = 0.05 and power (1 − *β*) = 0.95, the required sample size is approximately *N* ≈ 146. The final sample of *N* = 208 therefore exceeds these thresholds, indicating sufficient power to detect small–to–medium associations between 24-hour movement behaviors and perceived stress under commonly used effect-size assumptions.

### 2.2 Measurement of 24-hour movement behaviors

Participants were instructed to wear an ActiGraph wGT3X-BT (Actigraphcorp, Pensacola, USA) triaxial accelerometer on their right hip for 24 h a day for seven consecutive days. While accelerometers can estimate sleep, we additionally cross-validated participants’ sleep duration using daily sleeping and waking times recorded via the Pittsburgh Sleep Quality Index questionnaire as sleep log for more accurate estimation. The accelerometer was set to record data at 100 Hz, later aggregated into 5-s epochs. Participants could remove the device during water-based activities (e.g., swimming) or other circumstances (e.g., contact sports) but were asked to reattach it afterward. Data were processed using ActiLife 6.13.3 software (Actigraphcorp, Pensacola, USA). Activity intensity thresholds were defined as: SB = 0–99 counts per minute (cpm), LPA = 100–1951 cpm, moderate physical activity = 1952–5,724 cpm, and vigorous physical activity ≥5,725 cpm (Freedson et al., 1998). This method was chosen to maintain consistency in data collection. Valid days were defined as ≥10 h of wear time during waking hours, and participants needed at least three valid days for inclusion. Non-wear time was defined as ≥60 consecutive minutes of zero cpm.

### 2.3 Perceived stress measurement

The Perceived Stress Scale (PSS-14) assessed perceived stress. PSS-14 is a widely used 14-item self-report instrument recognized for its reliability, validity, and cost-effectiveness (Huang F. et al., 2020). Participants responded using a five-point Likert scale ranging from 0 (never) to 4 (very often), indicating how often they experienced stress-related events (e.g., “In the last month, how often have you felt that you were unable to control important things in your life?”). Items 4, 5, 7, 9, 10, and 13 were reverse-coded, and all items were summed for a total score (range: 0–56). Higher scores indicate greater perceived stress. In this study, internal consistency was good (Cronbach’s alpha = 0.78).

### 2.4 Covariates

Based on previous research, covariates included age (years), gender (male/female), body mass index [BMI; calculated from self-reported height (cm) and weight (kg)], and parental education level (junior high school or below, high school, university, master’s degree or above). These factors were controlled for in the regression analyses.

### 2.5 Statistical analysis

All continuous variables are presented as mean ± standard deviation (SD), and categorical variables as frequencies and percentages (*n*, %). Sleep, SB, LPA, and MVPA were treated as a four-part composition summing to 24 h. Compositional means (geometric means) described central tendencies, and a variation matrix depicted dispersion: lower variance between pairs indicated stronger proportional co-dependence. Using the *compositions* package in R (version 2.0–4), compositional data were transformed with isometric log-ratio (ILR) to map the simplex space into Euclidean space. After controlling for confounders, compositional multiple linear regression was performed using all ILR coordinates of each behavior to examine associations with perceived stress.

Based on the fitted model, isotemporal substitution analysis predicted changes in perceived stress when time was reallocated among behaviors. Standardized regression coefficients (*β*) and 95% confidence intervals (CI) were reported.

## 3 Results

### 3.1 Participant characteristics

A total of 208 university students were included, with a mean age of 20.23 ± 1.09 years. Gender distribution was nearly balanced, 98 males (47.1%) and 110 females (52.9%). BMI classification showed 161 participants (77.4%) had normal weight, 29 (13.9%) were overweight, and 18 (8.6%) were obese. The mean perceived stress score was 25.07 ± 6.08, indicating a moderate stress level. Participant characteristics are presented in Table 1.

**Table 1:** Basic characteristics of participants (*N* = 208).

Characteristic	*n* (%) / Mean ± SD
Age (years)	20.23 ± 1.09
Gender	
Male	98 (47.1%)
Female	110 (52.9%)
BMI category	
Normal	161 (77.4%)
Overweight	29 (13.9%)
Obese	18 (8.6%)
Father’s education	
High school or lower	137 (64.8%)
Undergraduate degree	61 (30.3%)
Graduate degree or above	10 (4.9%)
Mother’s education	
High school or lower	142 (68.6%)
Undergraduate degree	59 (28.4%)
Graduate degree or above	7 (3.0%)
Perceived stress score	25.07 ± 6.08

### 3.2 Distribution of 24-hour movement behaviors

**Figure 2:**
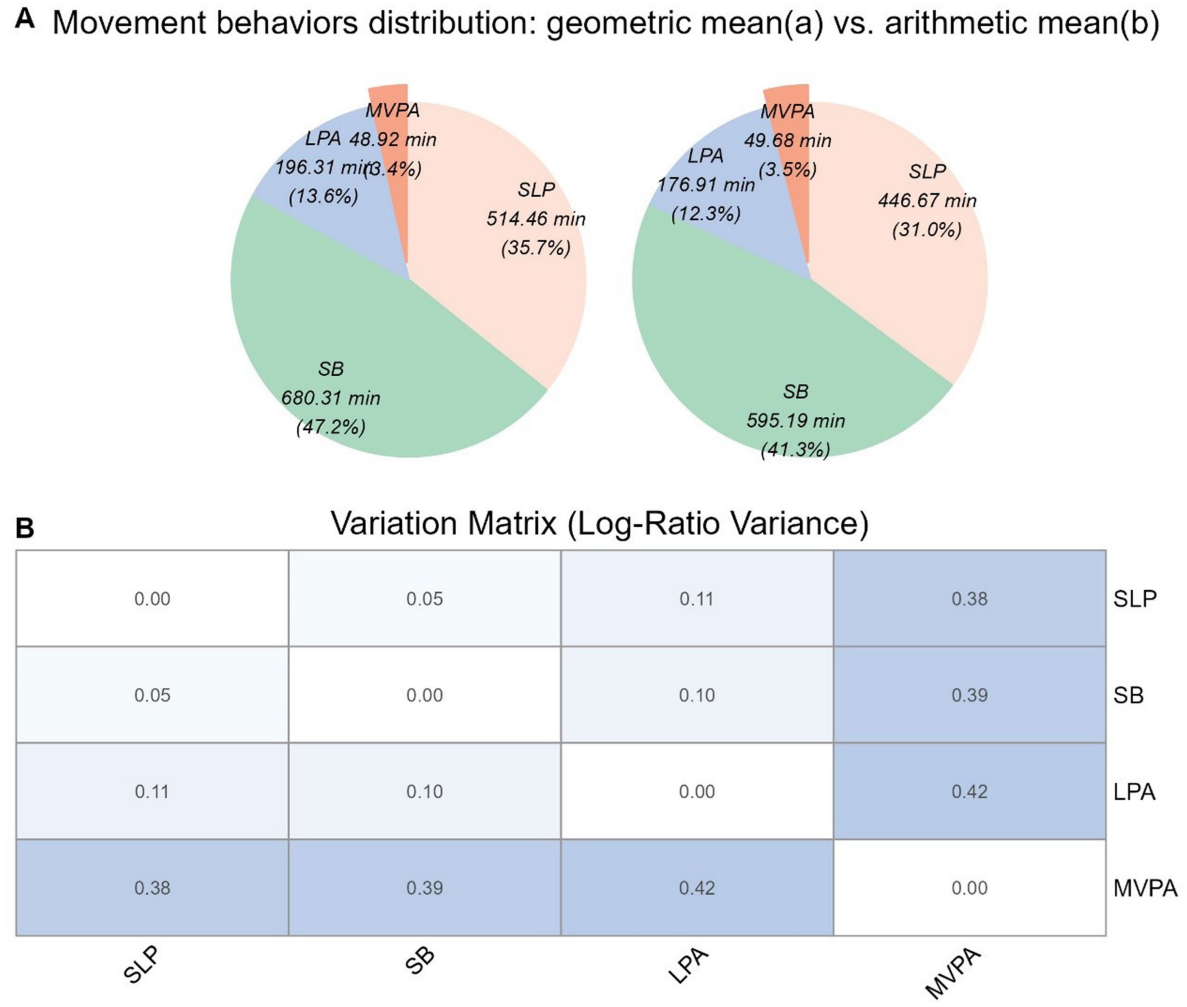
**(A)** Movement behaviors distribution based on geometric mean (a) and arithmetic mean (b). The pie charts display the proportion of time spent in each behavior: MVPA (moderate-to-vigorous physical activity), LPA (light physical activity), SB (sedentary behavior), and SLP (sleep). **(B)** Variation matrix of log-ratio variances. This matrix shows the variance between pairs of behaviors, where lower values indicate stronger co-dependence.

Geometric means indicated that MVPA, LPA, SB, and sleep accounted for 48.92 min/day (3.39%), 196.31 min/day (13.63%), 680.30 min/day (47.24%), and 514.46 min/day (35.72%), respectively. SB had the largest proportion, while MVPA was the smallest. The variation matrix revealed the lowest log-ratio variance between SB and sleep, indicating the strongest co-dependence and easiest substitution, whereas LPA and MVPA had the highest variance, suggesting the weakest co-dependence and hardest substitution (Figure 2).

### 3.3 Association between 24-hour movement behaviors and perceived stress

Compositional linear regression analysis revealed distinct associations between daily activity behaviors and perceived stress (Table 2). The overall model explained 17% of the variance in perceived stress (*R*^2^ = 0.17, Adjusted *R*^2^ = 0.14). Time spent in MVPA was significantly and inversely associated with perceived stress (*β* = −2.25, *SE* = 0.816, *t* = −2.765, *p* = 0.006), indicating that a greater proportion of MVPA relative to other behaviors was associated with lower stress levels. Similarly, LPA was also inversely related to perceived stress (*β* = −3.39, *SE* = 1.681, *t* = −2.021, *p* = 0.044). In contrast, SB showed a strong positive association with perceived stress (*β* = 7.95, *SE* = 2.425, *t* = 3.278, *p* = 0.001), suggesting that a higher proportion of sedentary time was linked to greater perceived stress. Sleep was not significantly associated with perceived stress (*β* = −2.29, *SE* = 2.337, *t* = −0.983, *p* = 0.327).

**Table 2:** Association between 24-h movement behaviors and perceived stress.

Behavior	*β*	SE	*t* value	*p* value	*R* ^2^	Adjusted *R*^2^
ILR/ln (MVPA: geometric mean of remaining behaviors)	−2.25	0.816	−2.765	0.006	0.17	0.14
ILR/ln (LPA: geometric mean of remaining behaviors)	−3.39	1.681	−2.021	0.044		
ILR/ln (SLP: geometric mean of remaining behaviors)	−2.29	2.337	−0.983	0.327		
ILR/ln (SB: geometric mean of remaining behaviors)	7.95	2.425	3.278	0.001		

*β* = unstandardized regression coefficient for the first pivot coordinate. *β* represents the change in perceived stress score associated with variation in each behavior relative to the other three behaviors. SE, standardized error; MVPA, moderate-to-vigorous physical activity; LPA, light physical activity, SB, sedentary behavior; SLP, sleep.

### 3.4 Time reallocation between different behaviors

**Figure 3:**
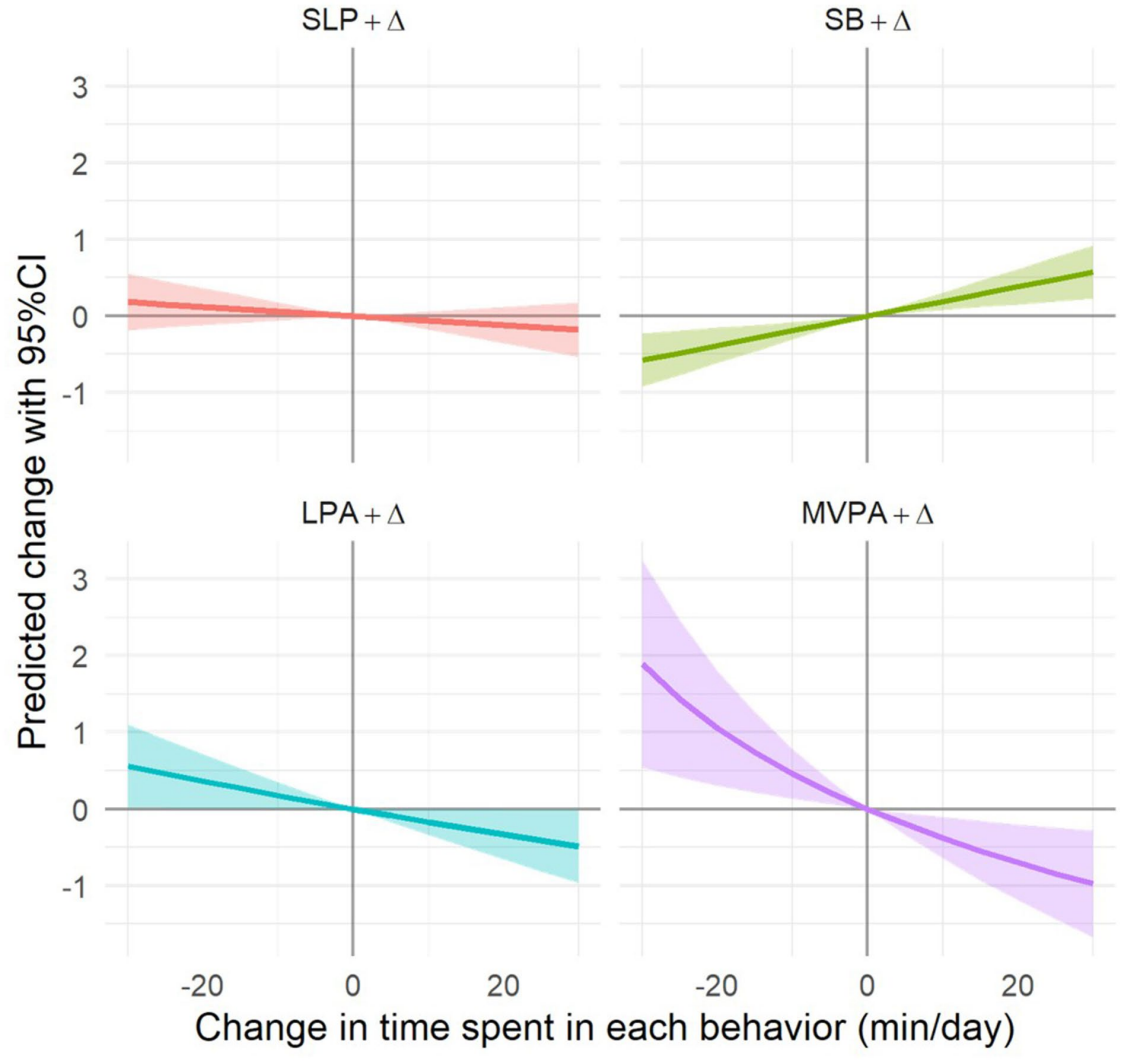
Dose–response relationships of time reallocation between different behaviors. MVPA, moderate-to-vigorous physical activity; LPA, light physical activity, SB, sedentary behavior; SLP, sleep.

The isotemporal substitution analysis (Table 3) revealed that reallocating 30 min of time between different behaviors was associated with changes in perceived stress scores. Substituting 30 min of SB with MVPA was associated with the largest reduction in perceived stress (*β* = −1.245, 95% CI: −1.955 to −0.535, *p* < 0.05). Similarly, replacing SB with LPA (*β* = −0.728, 95% CI: −1.240 to −0.217, *p* < 0.05) or sleep (*β* = −0.423, 95% CI: −0.806 to −0.039, *p* < 0.05) also associated with significantly lower stress scores, though the magnitude of reduction was smaller. Conversely, replacing 30 min of MVPA with SB was associated with an increase in perceived stress by 2.153 points (95% CI: 0.799 to 3.507, *p* < 0.05), while replacing LPA with SB was associated with an increase in stress by 0.785 points (95% CI: 0.214 to 1.355, *p* < 0.05), and replacing SLP with SB was associated with an increase in stress by 0.416 points (95% CI: 0.027 to 0.806, *p* < 0.05). Additionally, substituting MVPA with LPA (*β* = 1.438, 95% CI: 0.006 to 2.870, *p* < 0.05) or sleep (*β* = 1.743, 95% CI: 0.364 to 3.123, *p* < 0.05) associated with a significant increase in stress, suggesting that reducing MVPA was associated with higher stress. The dose–response relationships of time reallocation between different behaviors are illustrated in Figure 3.

**Table 3:** The result of time reallocation between different behaviors.

	Isotemporal substitution model			
	MVPA ↑	LPA ↑	SLP ↑	SB ↑
MVPA ↓	–	1.438 (0.006, 2.870)*	1.743 (0.364, 3.123)*	2.153 (0.799, 3.507)*
LPA ↓	−0.446 (−1.311, 0.417)	–	0.375 (−0.204, 0.955)	0.785 (0.214, 1.355)*
SLP ↓	−0.815 (−1.559, −0.071)*	−0.298 (−0.825, 0.227)	–	0.416 (0.027, 0.806)*
SB ↓	−1.245 (−1.955, −0.535)*	−0.728 (−1.240, −0.217)*	−0.423 (−0.806, −0.039)*	–

Values are predicted changes in perceived stress score (95% CI). *p < 0.05. ↑ indicates a 30 min increase in the behavior, ↓ indicates a 30 min decrease in the behavior. MVPA, moderate-to-vigorous physical activity; LPA, light physical activity, SB, sedentary behavior; SLP, sleep.

### 3.5 Time proportion change for each behavior

**Figure 4:**
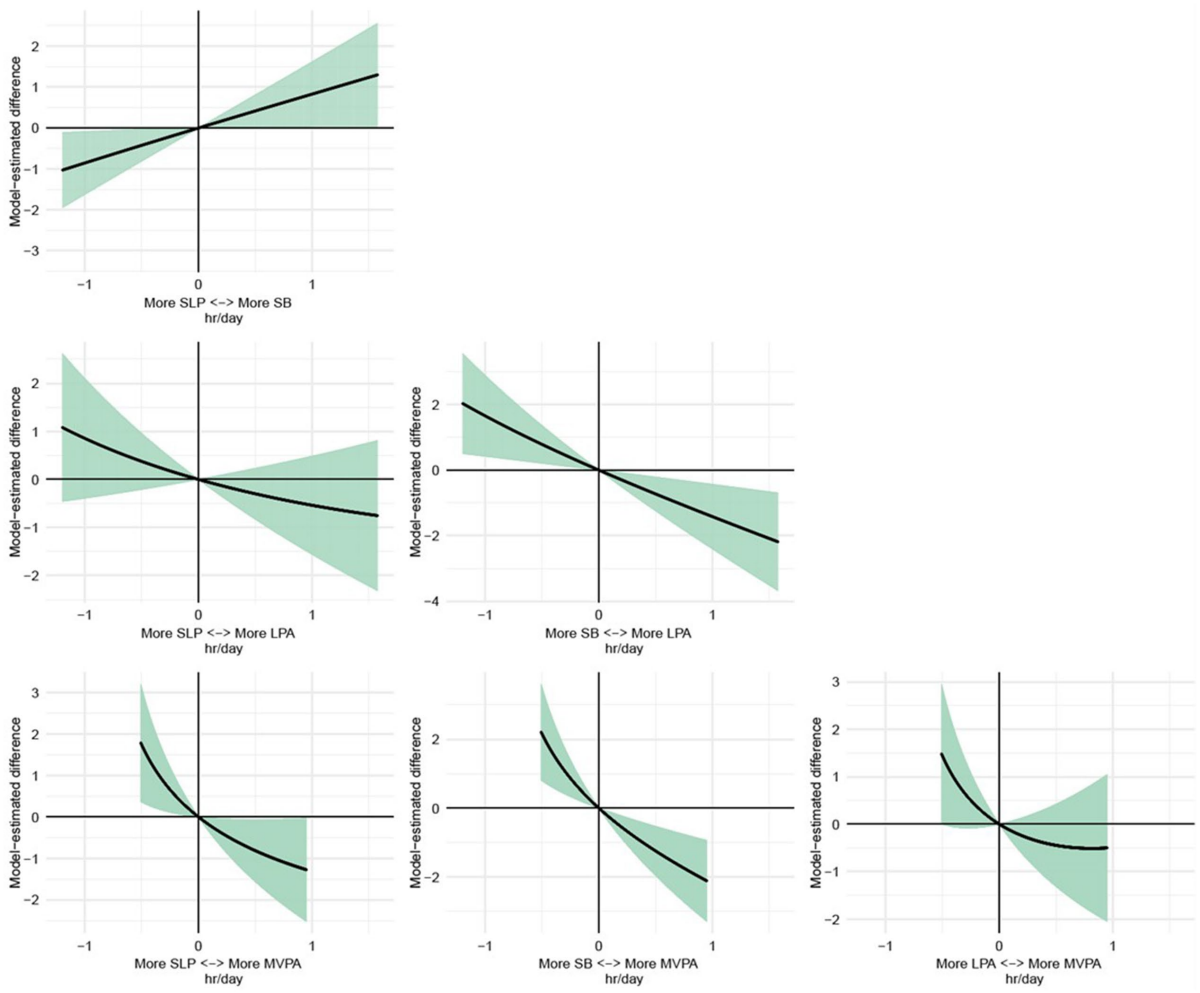
Dose–response relationships of proportional substitution for each behavior. MVPA, moderate-to-vigorous physical activity; LPA, light physical activity, SB, sedentary behavior; SLP, sleep.

The proportional substitution analysis examined the effect of changing the relative share of each behavior by 10% of the total 24-hour day (Table 4). Increasing MVPA by 10% (approximately 5 min/day) was associated with a significant reduction in perceived stress (*β* = −0.197, 95% CI: −0.337 to −0.067). Conversely, decreasing MVPA by 10% was associated with an increase in perceived stress (*β* = 0.217, 95% CI: 0.041–0.393). Similarly, a 10% increase in LPA (approximately 20 min/day) was linked to a significant decline in stress (*β* = −0.333, 95% CI: −0.658 to −0.008), whereas a 10% reduction in LPA was associated with higher stress levels (*β* = 0.363, 95% CI: 0.009–0.717). For sleep, neither a 10% increase (*β* = −0.306, 95% CI: −0.922 to 0.309) nor a 10% decrease (*β* = 0.320, 95% CI: −0.323 to 0.964) showed significant associations with perceived stress. In contrast, changes in SB had the most pronounced effects. A 10% increase in SB time (approximately 68 min/day) was strongly associated with higher perceived stress (*β* = 1.301, 95% CI: 0.518–2.085), while a 10% reduction in SB corresponded to a significant stress reduction (*β* = −1.315, 95% CI: −2.107 to −0.523). The dose–response relationships of proportional substitution for each behavior are illustrated in Figure 4.

**Table 4:** The results of the time proportion change for each behavior.

Behavior	10% of time (min)	Predicted change (+10%)	Predicted change (−10%)
MVPA	5	−0.197 (−0.337, −0.067)	0.217 (0.062, 0.373)
LPA	20	−0.333 (−0.658, −0.008)	0.363 (0.009, 0.717)
SLP	52	−0.306 (−0.922, 0.309)	0.320 (−0.323, 0.964)
SB	68	1.301 (0.518 2.085)	−1.315 (−2.107, −0.523)

MVPA, moderate-to-vigorous physical activity; LPA, light physical activity, SB, sedentary behavior; SLP, sleep.

## 4 Discussion

This study used objectively measured movement behavior data and compositional data analysis to explore the relationship between 24-hour movement behaviors and perceived stress in university students. Using compositional regression and isotemporal substitution models, we systematically evaluated the role of MVPA, LPA, SB, and sleep in stress perception. The findings showed that MVPA and LPA were significantly negatively associated with perceived stress, whereas SB was positively associated, and sleep showed no significant relationship. Substitution analysis further revealed that replacing SB with MVPA, LPA, or sleep was associated with significantly reduced stress, whereas SB displacing other activities was linked to increased stress. These findings highlight the potential links between time allocation in a fixed 24-hour period and mental health, suggesting that redistributing time among behaviors is closely tied to perceived stress.

Our analyses indicated that daily MVPA and LPA were inversely associated with perceived stress. This aligns with extensive previous research. Chronic stress can cause physiological and psychological fatigue, while physical activity provides varied stimulation that is linked to improved regulation of brain function. Numerous longitudinal and intervention studies have confirmed the protective effects of regular physical activity on mental health. Physiologically, physical activity regulates hypothalamic–pituitary–adrenal (HPA) axis responses and promotes the release of neurotransmitters such as dopamine and serotonin, which are critical for emotional regulation and alleviating depression and anxiety (Schoenfeld et al., 2013; Kim et al., 2018; Mennitti et al., 2024). Endorphins, known as “happiness hormones,” are released during exercise, and have been linked to the relief of stress and unpleasant feelings (Yau et al., 2011; Li et al., 2019). Physical activity is also associated with improved cardiovascular responses, such as lower blood pressure and heart rate (Huang et al., 2013; Franklin et al., 2021). Psychologically, physical activity is a potent intervention that has been associated with a reduction in repetitive negative thinking, such as rumination and worry. It is not only a form of recreation but can also enhance cognitive control. In addition, physical activity can improve self-esteem and body image, and may mitigate stress by building psychological resources through the cultivation of psychological resilience (VanKim and Nelson, 2013; Zhang et al., 2024; Liu and Liu, 2025; Wang et al., 2025). Social interaction is another key stress-buffering mechanism; team-based activities such as football are particularly effective for stress reduction because they provide a sense of belonging and social support (Andersen et al., 2019). Notably, this study also found that LPA was associated with lower stress, contrasting the traditional focus solely on MVPA. LPA includes walking, light household chores, and slow cycling—activities that are easier to integrate into daily life and have higher adherence. Although evidence regarding the mental health benefits of LPA remains inconclusive, some studies have shown that lifestyle LPA interventions are associated with effective stress reduction. Recent evidence indicates that even LPA is associated with mental health benefits, particularly for those who are insufficiently active (Füzéki et al., 2017; Felez-Nobrega et al., 2021). For individuals who are sedentary and experiencing high stress, substituting SB with LPA may represent an important first step toward improving mental health.

We also observed a significant positive relationship between SB and stress. This finding is consistent with global research linking SB to both metabolic risks and mental health issues (Zhai et al., 2015; Lee and Kim, 2018; Jiang et al., 2020). Prolonged SB may be linked to hindered stress relief and has been associated with increased heart rate, impaired heart rate variability, and blunted cortisol responses (Chauntry et al., 2022). Mechanistically, SB often involves passive information intake, reduced social interaction, and disrupted circadian rhythms, collectively affecting emotional regulation and mental health (Teychenne et al., 2019). SB’s high proportion may indirectly increase stress by displacing active time, aligning with the concept of “time reallocation” in health outcomes (Wang S. et al., 2023; Chandler et al., 2024). These physiological and psychological mechanisms likely contribute to higher perceived stress. Thus, reducing SB and maintaining moderate activity and rest are important strategies associated with the mitigation of SB’s negative health effects. Substitution analysis reinforced that replacing SB with MVPA or LPA was associated with reduced stress, suggesting SB is not merely the inverse of physical activity but an independent health risk behavior.

Regarding sleep, this study found no statistically significant association between sleep duration and stress. First, this may be due to bias arising from the use of self-reported sleep duration assessment methods. It is recommended that future studies employ objective sleep measurement methods. Second, we speculate this may relate to the “U-shaped” relationship between sleep and health: only moderate sleep duration is associated with health benefits, while excessively short or long sleep is linked to an increased disease risk (Wang et al., 2024). Thus, a non-linear relationship between sleep and stress may exist, which our linear models could not detect. Third, sleep quality, rather than duration, may have a stronger link to stress (Castiglione-Fontanellaz et al., 2023). Moreover, among university students, “social jetlag,” the mismatch between weekday and weekend sleep timing, which is a highly relevant factor that can lead to circadian disruption and psychological distress, aspects that sleep duration alone cannot capture (Yang et al., 2023). Furthermore, isotemporal substitution results suggested that replacing SB with sleep or other active behaviors significantly lowered stress. This implies that while sleep duration was not directly associated with stress in our study, optimizing the balance between sleep and other behaviors is still associated with benefits for stress reduction (Rosenberger et al., 2019). Overall, this study’s compositional data analysis and isotemporal substitution models provided a comprehensive perspective, revealing the complementarity and proportional distribution of daily activities and their effects on perceived stress. Specifically, reducing SB and reallocating time to MVPA, LPA, or even extending sleep appropriately is associated with a restoration of perceived stress levels.

These findings have important public health implications, reinforcing the 24-hour movement and health guidelines, which advocate considering the interplay between multiple behaviors rather than focusing on single activities (e.g., only counting daily MVPA minutes). From a practical standpoint, the stress reduction from replacing 30 min of SB with MVPA, while statistically modest, could represent a meaningful shift for a student, potentially moving them from a moderate to a lower stress category. This highlights that even small, manageable changes in daily routines can have a tangible impact on well-being and may translate to improved daily functioning and academic concentration. By accounting for time reallocations among all daily activities, health interventions may maximize benefits (Groves et al., 2024). A concrete intervention could be the introduction of short “movement breaks” into university lectures. Research shows that 5–10 min movement breaks are associated with reduced sedentary time and improved attention and enjoyment of learning, without adversely affecting academic performance (Peiris et al., 2021). Additionally, drawing on principles from behavioral economics, framing an activity as enjoyable and social rather than as a chore can increase its uptake (Zimmerman, 2009).

## 5 Limitations

Several factors should be considered when interpreting these findings. First, this cross-sectional design precludes causal inference, even though isotemporal substitution analyses suggested significant associations between time reallocations and stress. Future studies should adopt longitudinal or intervention designs to validate causality. Second, the generalizability of our findings is limited. The use of convenience sampling and a sample drawn from a single location during a single season may not be representative of the broader university student population. Caution is needed when extending these findings to other cultural contexts, and further verification through cross-cultural research is warranted. Third, several measurement limitations should be noted. Sleep duration was based on self-report, which may introduce measurement bias and could explain the non-significant relationship found between sleep and stress. Fourth, this study treated SB as a single indicator. We recommend that future research use accelerometer data to examine SB patterns, such as the duration of sedentary bouts (continuous, uninterrupted periods) and the frequency of sedentary breaks. Evidence suggests that frequent interruptions and shorter sedentary bouts are associated with better health outcomes, whereas prolonged, uninterrupted sedentary time may be particularly detrimental to mental health (Helgadóttir et al., 2015). Fifth, not all sedentary time is the same. We recommend distinguishing between mentally passive SB (e.g., watching television) and mentally active SB (e.g., studying). Studies have shown that mentally passive SB is more strongly associated with depression and anxiety than mentally active SB (Huang Y. et al., 2020; Huang T. et al., 2022). Moreover, we did not measure potential confounding variables such as academic burden, socioeconomic status, or dietary habits (e.g., caffeine intake), which future research should consider including. Additionally, our criterion for valid accelerometer data (at least 3 days) may be relatively lenient, and future studies could employ stricter standards to verify the results. Finally, the analytical approach itself has inherent limitations. The results of compositional data analysis are inherently relative, meaning the association of one behavior is interpreted in relation to all other behaviors in the composition. This requires careful consideration during interpretation.

## 6 Conclusion

This study indicates that optimizing the allocation of 24-hour movement behaviors is significantly associated with perceived stress in Chinese university students. Reallocating time from SB to MVPA or LPA is linked to lower levels of perceived stress. While the observed effect sizes are modest, they suggest that practical interventions promoting such behavioral substitutions, such as integrating short “movement breaks” into the academic day, are promising for stress management. It is important to acknowledge that the null finding for sleep may be influenced by methodological limitations. Future public health guidelines should consider the entire 24-hour day. Crucially, longitudinal and intervention studies are needed to establish causality and confirm the long-term mental health benefits of these behavioral modifications.

## Data Availability

The raw data supporting the conclusions of this article will be made available by the authors, without undue reservation.

## References

[ref1] Andersen M. H. Ottesen L. Thing L. F. (2019). The social and psychological health outcomes of team sport participation in adults: an integrative review of research. Scand. J. Public Health. 47, 832–850. doi: 10.1177/1403494818791405, PMID: 30113260

[ref2] Badger J. Quatromoni P. A. Morrell J. S. (2019). Relationship between stress and healthy lifestyle factors of college students. Health Behav. Policy Rev. 6, 43–55. doi: 10.14485/HBPR.6.1.4

[ref3] Castiglione-Fontanellaz C. E. G. Schaufler S. Wild S. Hamann C. Kaess M. Tarokh L. (2023). Sleep regularity in healthy adolescents: associations with sleep duration, sleep quality, and mental health. J. Sleep Res. 32:e13865. doi: 10.1111/jsr.13865, PMID: 36852716

[ref4] Chandler M. C. Ellison O. K. McGowan A. L. Fenn K. M. Pontifex M. B. (2024). Physical activity and sleep moderate the relationship between stress and screen time in college-aged adults. J. Am. Coll. Heal. 72, 1401–1411. doi: 10.1080/07448481.2022.2077110 35613432

[ref5] Chauntry A. J. Bishop N. C. Hamer M. Paine N. J. (2022). Sedentary behaviour, physical activity and psychobiological stress reactivity: a systematic review. Biol. Psychol. 172:108374. doi: 10.1016/j.biopsycho.2022.108374, PMID: 35667480

[ref6] Dumuid D. Pedišić Ž. Stanford T. E. Martín-Fernández J. A. Hron K. Maher C. A.. (2019). The compositional isotemporal substitution model: a method for estimating changes in a health outcome for reallocation of time between sleep, physical activity and sedentary behaviour. Stat. Methods Med. Res. 28, 846–857. doi: 10.1177/0962280217737805, PMID: 29157152

[ref7] Dumuid D. Stanford T. E. Martin-Fernández J. A. Pedišić Ž. Maher C. A. Lewis L. K.. (2018). Compositional data analysis for physical activity, sedentary time and sleep research. Stat. Methods Med. Res. 27, 3726–3738. doi: 10.1177/0962280217710835, PMID: 28555522

[ref8] Felez-Nobrega M. Bort-Roig J. Ma R. Romano E. Faires M. Stubbs B.. (2021). Light-intensity physical activity and mental ill health: a systematic review of observational studies in the general population. Int. J. Behav. Nutr. Phys. Act. 18:123. doi: 10.1186/s12966-021-01196-7, PMID: 34526048 PMC8444599

[ref9] Firth J. Solmi M. Wootton R. E. Vancampfort D. Schuch F. B. Hoare E.. (2020). A meta-review of “lifestyle psychiatry”: the role of exercise, smoking, diet and sleep in the prevention and treatment of mental disorders. World Psychiatry. 19, 360–380. doi: 10.1002/wps.20773, PMID: 32931092 PMC7491615

[ref10] Franklin B. A. Rusia A. Haskin-Popp C. Tawney A. (2021). Chronic stress, exercise and cardiovascular disease: placing the benefits and risks of physical activity into perspective. Int. J. Environ. Res. Public Health. 18:9922. doi: 10.3390/ijerph18189922, PMID: 34574843 PMC8471640

[ref11] Freedson P. S. Melanson E. Sirard J. (1998). Calibration of the computer science and applications, Inc. accelerometer. Med. Sci. Sports Exerc. 30, 777–781. doi: 10.1097/00005768-199805000-00021, PMID: 9588623

[ref12] Füzéki E. Engeroff T. Banzer W. (2017). Health benefits of light-intensity physical activity: a systematic review of accelerometer data of the national health and nutrition examination survey (NHANES). Sports Med. 47, 1769–1793. doi: 10.1007/s40279-017-0724-0, PMID: 28393328

[ref13] Ge Y. Xin S. Luan D. Zou Z. Bai X. Liu M.. (2020). Independent and combined associations between screen time and physical activity and perceived stress among college students. Addict. Behav. 103:106224. doi: 10.1016/j.addbeh.2019.106224, PMID: 31862620

[ref14] Gerber M. Ludyga S. Mücke M. Colledge F. Brand S. Pühse U. (2017). Low vigorous physical activity is associated with increased adrenocortical reactivity to psychosocial stress in students with high stress perceptions. Psychoneuroendocrinology 80, 104–113. doi: 10.1016/j.psyneuen.2017.03.004, PMID: 28324699

[ref15] Groves C. I. Huong C. Porter C. D. Summerville B. Swafford I. Witham B.. (2024). Associations between 24-hour movement behaviors and indicators of mental health and well-being across the lifespan: a systematic review. J. Act. Sedentary Sleep Behav. 3:9. doi: 10.1186/s44167-024-00048-6, PMID: 40217439 PMC11960375

[ref16] Gu X. Mao E. (2023). The impacts of academic stress on college students’ problematic smartphone use and internet gaming disorder under the background of neijuan: hierarchical regressions with mediational analysis on escape and coping motives. Front. Psych. 13:1032700. doi: 10.3389/fpsyt.2022.1032700, PMID: 36683982 PMC9849911

[ref17] Helgadóttir B. Forsell Y. Ekblom Ö. (2015). Physical activity patterns of people affected by depressive and anxiety disorders as measured by accelerometers: a cross-sectional study. PLoS One 10:e0115894. doi: 10.1371/journal.pone.0115894, PMID: 25585123 PMC4293141

[ref18] Huang Z. Li J. (2025). The relationship between device-measured movement behaviors and optimal mental health in Chinese youth: a compositional data analysis. Ment. Health Phys. Act. 28:100664. doi: 10.1016/j.mhpa.2024.100664

[ref19] Huang Y. Li L. Gan Y. Wang C. Jiang H. Cao S.. (2020). Sedentary behaviors and risk of depression: a meta-analysis of prospective studies. Transl. Psychiatry 10:26. doi: 10.1038/s41398-020-0715-z, PMID: 32066686 PMC7026102

[ref20] Huang Z. Liu Y. Zhou Y. (2022). Sedentary behaviors and health outcomes among young adults: a systematic review of longitudinal studies. Healthcare 10:1480. doi: 10.3390/healthcare10081480, PMID: 36011137 PMC9408295

[ref21] Huang F. Wang H. Wang Z. Zhang J. Du W. Su C.. (2020). Psychometric properties of the perceived stress scale in a community sample of Chinese. BMC Psychiatry 20:130. doi: 10.1186/s12888-020-02520-4, PMID: 32197589 PMC7082906

[ref22] Huang C.-J. Webb H. E. Zourdos M. C. Acevedo E. O. (2013). Cardiovascular reactivity, stress, and physical activity. Front. Physiol. 4:314. doi: 10.3389/fphys.2013.00314 24223557 PMC3819592

[ref23] Huang T. Zheng K. Li S. Yang Y. Kong L. Zhao Y. (2022). Screen-based sedentary behaviors but not total sedentary time are associated with anxiety among college students. Front. Public Health 10:994612. doi: 10.3389/fpubh.2022.994612, PMID: 36339232 PMC9632443

[ref24] Jackson E. M. (2013). Stress relief: the role of exercise in stress management ACSM’s. Health Fit. J. 17, 14–19. doi: 10.1249/FIT.0b013e31828cb1c9

[ref25] Jiang L. Cao Y. Ni S. Chen X. Shen M. Lv H.. (2020). Association of sedentary behavior with anxiety, depression, and suicide ideation in college students. Front. Psych. 11:566098. doi: 10.3389/fpsyt.2020.566098, PMID: 33424653 PMC7793895

[ref26] Kim S.-Y. Jeon S. W. Shin D. W. Oh K. S. Shin Y. C. Lim S. W. (2018). Association between physical activity and depressive symptoms in general adult populations: an analysis of the dose-response relationship. Psychiatry Res. 269, 258–263. doi: 10.1016/j.psychres.2018.08.076, PMID: 30170283

[ref27] Kivimäki M. Bartolomucci A. Kawachi I. (2023). The multiple roles of life stress in metabolic disorders. Nat. Rev. Endocrinol. 19, 10–27. doi: 10.1038/s41574-022-00746-8, PMID: 36224493 PMC10817208

[ref28] Kivimäki M. Steptoe A. (2018). Effects of stress on the development and progression of cardiovascular disease. Nat. Rev. Cardiol. 15, 215–229. doi: 10.1038/nrcardio.2017.189, PMID: 29213140

[ref29] Lee E. Kim Y. (2018). Effect of university students’ sedentary behavior on stress, anxiety, and depression. Perspect. Psychiatr. Care 55:164. doi: 10.1111/ppc.12296 29797324 PMC7818186

[ref30] Li Q. Li L. Li C. Wang H. (2024). The association between moderate-to-vigorous physical activity and health-related quality of life in chinese adolescents: the mediating roles of emotional intelligence and perceived stress. Front. Psychol. 15:1477018. doi: 10.3389/fpsyg.2024.1477018, PMID: 39687563 PMC11646723

[ref31] Li A. Yau S. Y. Machado S. Wang P. Yuan T. F. So K. F. (2019). Enhancement of hippocampal plasticity by physical exercise as a polypill for stress and depression: a review. CNS Neurol. Disord. Drug Targets. 18, 294–306. doi: 10.2174/1871527318666190308102804, PMID: 30848219

[ref32] Liu X. Liu L. (2025). The mediating role of college students’ body image and self-esteem in the impact of physical activity on life satisfaction and mental health. Acta Psychol. 257:105103. doi: 10.1016/j.actpsy.2025.105103, PMID: 40446425

[ref33] McEwen B. S. (2008). Central effects of stress hormones in health and disease: understanding the protective and damaging effects of stress and stress mediators. Eur. J. Pharmacol. 583, 174–185. doi: 10.1016/j.ejphar.2007.11.071, PMID: 18282566 PMC2474765

[ref34] Mennitti C. Farina G. Imperatore A. de Fonzo G. Gentile A. la Civita E.. (2024). How does physical activity modulate hormone responses? Biomolecules 14:1418. doi: 10.3390/biom14111418, PMID: 39595594 PMC11591795

[ref35] Momen N. C. Plana-Ripoll O. Agerbo E. Benros M. E. Børglum A. D. Christensen M. K.. (2020). Association between mental disorders and subsequent medical conditions. N. Engl. J. Med. 382, 1721–1731. doi: 10.1056/NEJMoa1915784, PMID: 32348643 PMC7261506

[ref36] Nowacka-Chmielewska M. Grabowska K. Grabowski M. Meybohm P. Burek M. Małecki A. (2022). Running from stress: neurobiological mechanisms of exercise-induced stress resilience. Int. J. Mol. Sci. 23:13348. doi: 10.3390/ijms232113348, PMID: 36362131 PMC9654650

[ref37] Pedišić Ž. Dumuid D. Olds T. (2017). Integrating sleep, sedentary behaviour, and physical activity research in the emerging field of time-use epidemiology: definitions, concepts, statistical methods, theoretical framework, and future directions. Kinesiology 49, 252–269.

[ref38] Peiris C. L. O’Donoghue G. Rippon L. Meyers D. Hahne A. de Noronha M.. (2021). Classroom movement breaks reduce sedentary behavior and increase concentration, alertness and enjoyment during university classes: a mixed-methods feasibility study. Int. J. Environ. Res. Public Health 18:5589. doi: 10.3390/ijerph18115589, PMID: 34073761 PMC8197210

[ref39] Rosenberger M. E. Fulton J. E. Buman M. P. Troiano R. P. Grandner M. A. Buchner D. M.. (2019). The 24-hour activity cycle: a new paradigm for physical activity. Med. Sci. Sports Exerc. 51, 454–464. doi: 10.1249/MSS.0000000000001811, PMID: 30339658 PMC6377291

[ref40] Schoenfeld T. J. Rada P. Pieruzzini P. R. Hsueh B. Gould E. (2013). Physical exercise prevents stress-induced activation of granule neurons and enhances local inhibitory mechanisms in the dentate gyrus. J. Neurosci. Off. J. Soc. Neurosci. 33, 7770–7777. doi: 10.1523/JNEUROSCI.5352-12.2013, PMID: 23637169 PMC3865561

[ref41] Stults-Kolehmainen M. A. Sinha R. (2014). The effects of stress on physical activity and exercise. Sports Med. 44, 81–121. doi: 10.1007/s40279-013-0090-5, PMID: 24030837 PMC3894304

[ref42] Tan S. L. Jetzke M. Vergeld V. Müller C. (2020). Independent and combined associations of physical activity, sedentary time, and activity intensities with perceived stress among university students: internet-based cross-sectional study. JMIR Public Health Surveill. 6:e20119. doi: 10.2196/20119, PMID: 33174855 PMC7688394

[ref43] Tavolacci M. P. Ladner J. Grigioni S. Richard L. Villet H. Dechelotte P. (2013). Prevalence and association of perceived stress, substance use and behavioral addictions: a cross-sectional study among university students in France, 2009–2011. BMC Public Health. 13:724. doi: 10.1186/1471-2458-13-724, PMID: 23919651 PMC3750571

[ref44] Teychenne M. Stephens L. D. Costigan S. A. Olstad D. L. Stubbs B. Turner A. I. (2019). The association between sedentary behaviour and indicators of stress: a systematic review. BMC Public Health. 19:1357. doi: 10.1186/s12889-019-7717-x, PMID: 31647002 PMC6813058

[ref45] VanKim N. A. Nelson T. F. (2013). Vigorous physical activity, mental health, perceived stress, and socializing among college students. Am. J. Health Promot. 28, 7–15. doi: 10.4278/ajhp.111101-QUAN-395, PMID: 23470187 PMC3758412

[ref46] Wang S. Liang W. Song H. Su N. Zhou L. Duan Y.. (2023). Prospective association between 24-hour movement behaviors and mental health among overweight/obese college students: a compositional data analysis approach. Front. Public Health 11:1203840. doi: 10.3389/fpubh.2023.1203840, PMID: 37854249 PMC10579788

[ref47] Wang H. Liu Y. Zhang S. Xu Z. Yang J. (2023). Investigating links between moderate-to-vigorous physical activity and self-rated health status in adolescents: the mediating roles of emotional intelligence and psychosocial stress. Children 10:1106. doi: 10.3390/children10071106, PMID: 37508604 PMC10378217

[ref48] Wang S. Lu M. Dong X. Xu Y. (2025). Does physical activity-based intervention decrease repetitive negative thinking? A systematic review. PLoS One 20:e0319806. doi: 10.1371/journal.pone.0319806, PMID: 40168446 PMC11960971

[ref49] Wang F. Sun Z. Lin F. Xu Y. Wu E. Sun X.. (2024). Nonlinear relationships between sleep duration, mental health, and quality of life: the dangers of less sleep versus more sleep. Sleep Med. 119, 565–573. doi: 10.1016/j.sleep.2024.05.043, PMID: 38823335

[ref50] World Health Organization (2023) Stress. Available online at: https://www.who.int/news-room/questions-and-answers/item/stress (Accessed July 27, 2025).

[ref51] Yang F. N. Picchioni D. Duyn J. H. (2023). Effects of sleep-corrected social jetlag on measures of mental health, cognitive ability, and brain functional connectivity in early adolescence. Sleep 46:zsad259. doi: 10.1093/sleep/zsad259, PMID: 37788383 PMC10710981

[ref52] Yau S.-Y. Lau B. W.-M. So K.-F. (2011). Adult hippocampal neurogenesis: a possible way how physical exercise counteracts stress. Cell Transplant. 20, 99–111. doi: 10.3727/096368910X532846, PMID: 20887683

[ref53] Zhai L. Zhang Y. Zhang D. (2015). Sedentary behaviour and the risk of depression: a meta-analysis. Br. J. Sports Med. 49, 705–709. doi: 10.1136/bjsports-2014-093613, PMID: 25183627

[ref54] Zhang Y. Wen Z. Zhu Y. Guan G. (2024). Effects of physical exercise on body esteem among females: a meta-analysis. BMC Public Health 24:3387. doi: 10.1186/s12889-024-20861-7, PMID: 39639264 PMC11622481

[ref55] Zimmerman F. (2009). Using behavioral economics to promote physical activity. Prev. Med. 49, 289–291. doi: 10.1016/j.ypmed.2009.07.008, PMID: 19632266

